# Community palliative care services on addressing physical and psychosocial needs in people with advanced illness: a prospective cohort study

**DOI:** 10.1186/s12904-021-00840-0

**Published:** 2021-09-15

**Authors:** Helen Yue-lai Chan, Carmen Ka-man Chung, Shawn Sze-chai Tam, Rita Suk-kuen Chow

**Affiliations:** 1 The Nethersole School of Nursing, Faculty of Medicine, The Chinese University of Hong Kong, Shatin, Hong Kong SAR, China; 2 Endless Care Services, Elderly Services Section, Tung Wah Group of Hospitals, Kwun Tong, Hong Kong SAR, China

**Keywords:** Palliative care, Primary care, Communication, Community-based, Symptom burden, Quality of life

## Abstract

**Background:**

The integration of palliative care into primary health care has been advocated to improve its accessibility and the continuity of care. Recent studies on such an approach have mainly focused on health care cost and utilization. This study aims to evaluate the effects of a community interdisciplinary palliative care program on the symptom experience of patients with advanced disease.

**Methods:**

A prospective cohort study was conducted. The Integrated Palliative Care Outcome Scale was used for monthly assessment to monitor their condition. Wilcoxon signed-rank test was used to examine changes in symptom experience across time.

**Results:**

Forty-eight patients with a predominance of cancer diagnoses, enrolled in the program. They reported anxiety, hardly feeling at peace, and neither receiving information as wanted nor being able to share their feeling with family/friends as more overwhelming than physical symptoms. Improvements in emotional symptoms was statistically significant at 1-month follow up (*p* < 0.001). Improvements in communication/practical issues were also significant at the 1-month (*p* < 0.001) and 2-month (*p* = 0.005) follow-up. However, changes in symptom experiences in the subsequent months were not apparent.

**Conclusions:**

This study reveals the overwhelming emotional, communication and information needs among patients with advanced diseases and provides empirical evidence of the community palliative care program in short term. Further work is needed to strengthen the medical-social partnership to support care in place albeit health deterioration.

## Background

Palliative care aims to optimize quality of life among people with life-limiting conditions and their family through a holistic approach [1]. Evidence confirms that palliative care can mitigate pain and physical symptoms, attenuate psycho-spiritual distress, reduce avoidable hospital admission and length of stay, and prolong survival [2–6]. Given its positive impacts, the World Health Organization has asserted the need of integrating palliative care into primary health care to ensure wider and fair access to people with malignant conditions, progressive debilitating diseases or comorbidities in the community [6–8]. Multidisciplinary community-based palliative care services are available in a number of regions, including France, Germany, Belgium, Zimbabwe, Malaysia and Taiwan, to facilitate end-of-life care and home deaths [9, 10]. The key components included symptom assessment and management, psychosocial care, support to family and coordination of health and social services subject to individual needs. The care teams include but not limited to general practitioners, family physicians, community nurses and other allied health professionals to improve continuity of care between acute and home care [8, 11, 12]. Recent studies have focused on the effects of these services on healthcare utilization and cost, with limited attention paid toward patients’ outcomes [13, 14].

In Hong Kong, limited attention and resources have been devoted to supporting the development of community palliative care in Hong Kong, resulting in over-reliance on inpatient care [15, 16]. Over 90% of deaths in the older population and patients with advanced diseases occur in in-patient setting and one-third of them spent the last month of life in hospitals [16, 17]. Palliative care has been introduced in our community for nearly four decades. At present, a wide spectrum of services, including inpatient care, outpatient clinics, day care centers and home care services, was provided by the public healthcare services [16, 18]. Structured partnership between hospitals and community care sectors on community palliative care services in primary health care has not been established because home is not a preferred place of death due to cultural and legal barriers [18, 19]. There are concerns about troubles regarding police investigation and logistic arrangement of transportation of the deceased patient in the community, inadequate professional healthcare support for end-of-life care and fear of being regarded as haunted flat [17, 18]. Nevertheless, patients generally would like to be taken care at home till the last phase of life [17, 20]. To this end, the Hospital Authority has recently affirmed the importance of enhancing palliative care in the community settings as one of the strategic directions for healthcare service development [16].

To fill the service gap, a community-based palliative care project was initiated in 2014 by a non-government organization with the support of philanthropic funding. The organization has a long history of providing various kinds of subsidized community services in the local community. The project began in collaboration with three hospital palliative care units and later expanded in 2016 to receive referrals from all healthcare providers. Evaluation of the project in the early phase of development largely focused on satisfaction with care among patients and family members. This study aims to evaluate the effects of this project on patients’ symptom experience, including their physical symptoms and psychosocial concerns.

## Methods

### Intervention

The Community Palliative Care Support Project, entitled “Care and Love Companion”, aimed to provide home-based individualized multidisciplinary support to patients and their family members based on a medical-social collaboration model to optimize their quality of life. The project team consisted of a registered nurse and a social worker. Both of them were experienced in case management, medical and community care. The project services included comprehensive assessment using the Integrated Palliative Care Outcome Scale (IPOS), medication review, various non-pharmacological strategies for symptom management and health maintenance, such as aromatherapy, massage and oral supplements, home-based occupational therapy and dietitian consultation. The project team also provided psychosocial support through psychoeducation, counseling, advance care planning, coordination of financial, home care or funeral services, wish fulfilment and bereavement care, subject to individual care needs and resource availability. The team liaised with the hospital healthcare teams if any urgent medical follow up or medical investigation was needed. Subsequent home visits or phone follow up were arranged on need basis. During the COVID-19 pandemic, phone calls and video conferencing were the major means for the project team to maintain contact with the clients when home visits were not preferred. The support provided by the project continued until the patients died or withdrew from the project.

### Study design

A prospective cohort study was conducted to evaluate the project services based on the information routinely collected between October 2019 and December 2020.

### Participants

All patients who joined the project within the study period were included in the analysis, unless they did not provide consent for using their data for evaluation. The patients were referred by hospital healthcare teams and general practitioners if they noted that their patients have advanced condition using the surprise question that “I would not be surprised if this patient died in the next 12 months” and in need of community palliative care services. The exclusion criteria were patients aged under 18 years, lacked decision-making capacity to provide consent to join the project or refused to join the project.

### Measures

The Integrated Palliative Care Outcome Scale (IPOS) was used for assessing symptoms monthly via home visits or phone contacts in three domains: physical, emotional, and communication/ practical issues [21]. This scale is a valid and reliable patient reported outcome measure (PROM) for assessing the severity of symptoms and concerns due to advanced illness. Patients were asked to rate the severity of 13 physical symptoms, 4 emotional symptoms, and 3 items about communication or practical problems over the past week on a five-point Likert scale, from 0 to 5. Positively worded items, such as peace of mind, are reversely scored. So, a lower score suggests better experience. Given the limited access right to hospital records, information about healthcare utilization was obtained only through the self-report of patients or their family members.

### Data collection

The project was promoted to all medical and palliative care units in public hospitals and also through social media. Upon receiving a referral, either the project social worker or nurse contacted the patient through phone for eligibility screening and scheduled home visits within a week if they met the inclusion criteria. At the first home visit, the project team sought patient consent for using the data collected for evaluating the project services. They were reassured that no personal identifiers would be included in the findings and their participation in the study would not affect the services they received from the project. An information sheet about the nature and purpose of the evaluation study was given and written consent was obtained. Project evaluation was conducted by a research team independent of the project team. Participation in the project and the evaluation study was on voluntary basis. Patients who joined the project have the right to opt out from the evaluation. Assessments were conducted by the project team as part of the routine services on a monthly basis to identify the care needs of the patient and to monitor progress. They read aloud the questions on the IPOS and let the patients answer according to the response format. Interrater reliability between the ratings of the two project staff was assessed for the first five patients recruited, with over 90% of agreement achieved.

### Statistical analysis

SPSS version 26.0 (IBM Corp., Armonk, NY) was utilized for analysis. Descriptive statistics were used to present the demographic and the IPOS scores. The IPOS subscale scores were the sum of item scores within the domain. Only the scores in the first 6 months were used for analysis because the sample size left beyond this period of time was too small for meaningful analysis. Wilcoxon signed-rank test was applied to examine the changes in the IPOS subscale scores between two consecutive time points. A *p* value < 0.05 is considered statistically significant.

## Results

### Recruitment

A total of 79 patients were referred to the program within this research time frame. The research team successfully recruited 67 patients (84.8%) to the program, but 15 of them died before the first home visit. Twelve potential participants (15.2%) were excluded because they were too ill to give consent to join the program or readmitted and remained in the hospital, or there was no one answering the phone calls. Of the 52 patients approached, four (6.0%) patients or their family members declined the program. Eventually, 48 patients were enrolled in the program. Five (10.4%) of them withdrew from the study after enrollment. The major reasons of withdrawal include difficulties in speaking or listening (*n* = 2) and a feeling of stress (*n* = 1). The remainder did not provide a reason for their withdrawal (*n* = 2). The total number of home visit ranged from 1 to 7, with nearly two thirds (60.4%) receiving at least three home visits.

### Patients’ characteristics

Table 1 shows the patients’ characteristics. The mean age of the patients was 63.8 years, ranging from 28 to 94. Slightly more than half of them (54.2%) were younger than 65 years, and 24 participants (50%) were male. Over half (54.2%) was single, divorced or widowed. Two thirds (66.7%) were living with family members. The chief diagnosis of the patients was cancer, mainly lung (*n* = 9), colorectal (*n* = 5), liver (*n* = 5), and breast (*n* = 4) cancer, except for two with non-malignant diagnosis. Over half (52.1%) required assistance in self-care and four (8.3%) were bed bound. Twenty (41.7%) received home care support provided by the hospital palliative outreach teams which included nurses only, but none of them attended palliative care day care centers.

**Table 1:** Patients’ characteristics (*n* = 48)

	*n* (%)^a^
Mean age (SD)	63.8 (15.1)
Sex	
Male	24 (50.0)
Female	24 (50.0)
Marital status	
Single	13 (27.1)
Married	22 (45.8)
Widowed	9 (18.8)
Divorced	4 (8.3)
Living status	
Living alone	10 (20.8)
Living with maid only	5 (10.4)
Living with family	32 (66.7)
Care home	1 (2.1)
Chief diagnosis	
Cancer	46 (95.8)
Chronic renal failure	2 (4.2)
Self-care ability	
Independent	19 (39.6)
Need assistance	25 (52.1)
Bed bound	4 (8.3)
Experience of using hospital palliative care services	
Inpatient care	30 (62.5)
Day care center	0
Home care	20 (41.7)

^a^Number (percent), unless specified

### Symptom burden

At the baseline (Table 2), a considerable proportion of participants reported feeling anxious (79.2%), could hardly feel at peace (79.1%), could neither receive information as wanted (79.1%) nor share their feelings with family/friends (72.9%), and perceived practical matters as being unaddressed (77.1%). The ratings showed that these emotional symptoms and communication/practical issues were more overwhelming than the physical symptoms. Regarding physical symptoms, the three most distressing were weakness/lack of energy (70.8%), poor mobility (64.6%), and pain (52.1%).

**Table 2:** Symptom experience at baseline (*n* = 48)

	Prevalence	Rating^a^				
		Not at all (0)	Slight (1)	Moderate (2)	Severe (3)	Overwhelming/ All the time (4)
*Physical symptoms*						
• Pain	52.1	25.0	22.9	25.0	18.8	8.3
• Shortness of breath	25.0	41.7	33.3	14.6	6.3	4.2
• Weakness / Lack of energy	70.8	8.3	20.8	39.6	31.3	0
• Nausea	33.3	37.5	29.2	25.0	8.3	0
• Vomiting	14.6	75.0	10.4	12.5	2.1	0
• Poor appetite	45.8	27.1	27.1	25.0	18.8	2.1
• Constipation	10.4	79.2	10.4	6.3	4.2	0
• Sore / dry mouth	37.5	37.5	25.0	35.4	2.1	0
• Drowsiness	27.1	47.9	25.0	18.8	8.3	0
• Poor mobility	64.6	16.7	18.8	29.2	27.1	8.3
• Insomnia	36.4	54.5	9.1	30.3	6.1	0
• Oedema	12.1	72.7	15.2	3.0	6.1	3.0
• Dizziness	6.0	75.8	18.2	3.0	3.0	0
*Emotional symptoms*						
• Patient anxiety	79.2	10.4	10.4	37.5	31.3	10.4
• Family anxiety	68.8	18.8	12.5	29.2	22.9	16.7
• Depression	60.4	20.8	18.8	27.1	25.0	8.3
	Prevalence	Always (0)	Most of the time (1)	Sometimes (2)	Occasionally (3)	Not at all (4)
• Feeling at peace	79.1	8.3	12.5	35.4	35.4	8.3
*Communication/ Practical issues*						
• Sharing feelings	72.9	4.2	22.9	25.0	39.6	8.3
• Information	79.1	2.1	18.8	22.9	45.8	10.4
• Practical matters	77.1	2.1	20.8	52.1	20.8	4.2

Prevalence was defined as rating on the item at 2 or above

^a^Lower score better experience

### Changes over time

**Fig. 1:**
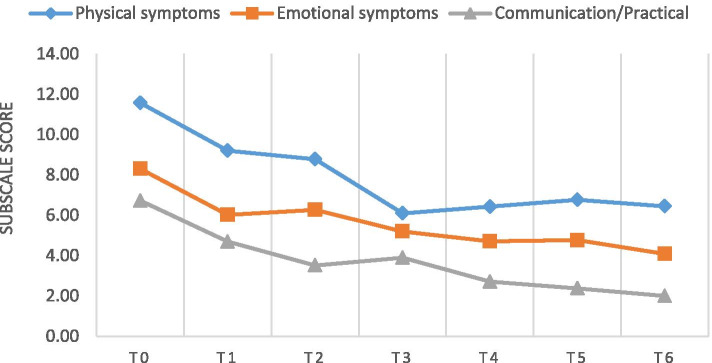
Changes in IPOS subscale mean scores over time

Figure 1 illustrates the changes in the IPOS subscale mean scores over 6 months. A decreasing trend in the mean scores was observed, suggesting improvement in various aspects among the patients. Pairwise comparisons of the IPOS subscale scores over time showed that improvement in emotional symptoms at the 1-month follow up was statistically significant (*p* < 0.001). Improvements in communication/ practical issues were also significant at the 1-month follow up (*p* < 0.001) and 2-month follow-up (*p* = 0.005).

### Healthcare utilization

The average number of hospital readmissions during the six-month study period was 3.52 (median = 2), with readmissions ranging from 0 to 14. Of the 20 patients who died within the study period, nine (45%) and eight (40%) were admitted to palliative care wards or hospices, respectively, for end-of-life care.

## Discussion

This study provides empirical evidence on the effects of a community-based palliative care program in the primary health care in Hong Kong. The patients in this project seems more seriously ill as the mortality rate was higher (41.7%) compared with those in other studies on community palliative care services (2.3 – 19.0%) [22, 23]. The most overwhelming symptoms reported by patients with advanced diseases at baseline were anxiety, could not feel at peace, unmet information needs and not able to share feelings with family or friends. Improvements in emotional symptoms and communication or practical issues among patients with advanced diseases were statistically significant, with moderate effect sizes, in the first 2 months of services. No significant differences were detected in symptom experience over the following months.

The high burden in communication and practical issues noted in our study appear as transcultural commonalities in patients and families when confronted with life closure. The patients initially reported a higher level of disturbance with emotional symptoms and concerns about communication and practical issues relative to physical symptoms. These findings were consistent with those using the same measurement tool in the United Kingdom [21]. Communication and practical issues were equally overwhelming as other distressing physical symptoms, such as weakness/lack of energy, poor mobility, and pain, herein and in the UK study. Unmet information needs and being unable to share feelings with others were particular prevalent concerns among palliative care patients in both regions [21]. Patients with life-limiting conditions often perceived a lack of informational support for advance care planning as the disease progresses and were unclear about the legitimacy of their concerns [24, 25]. This is likely because dysfunctional communication (such as avoidance, denial, and protective buffering) that prevents reciprocal disclosure of feelings and thoughts was common between cancer patients and their families [26]. The cultural taboo of discussing issues related to death in Chinese community further hinders the communication between patients, family members and healthcare providers on end-of-life care [27, 28].

The mix of expertise in the present interdisciplinary project was complementary to the existing nurse-led outreach service. The European Intersectorial and Multidisciplinary Palliative Care Research Training (EURO IMPACT) team has stressed the importance of involving different disciplines to deliver person-centered care in responding to psychosocial and information needs [29]. Likewise, person-centered care, including emotional support, providing reassurance and space for sharing personal worries, and an integrated team were highlighted from the perspectives of patients and family members as essential elements of community palliative care [11, 12, 25]. However, our findings concurred with other studies that the effects of community palliative care support were less apparent beyond the first 2 months [3, 22, 23]. Given that their psycho-spiritual distress and physical problems might elevate along the illness trajectory, from the stable to unstable and deteriorating phases [30], further work is needed to examine how to better support them as their conditions worsen.

One noteworthy point is that non-cancer patients are underrepresented in this project. Unlike other studies that focused on a specific non-cancer condition or that were supported by a particular palliative care team [3, 22], enrolment to this project was reliant on referral across care settings in the region. Clinicians’ hesitancy on prognostication has been identified as the major barrier for equitable access to palliative care among patients with non-cancer diagnoses [31]. Evidence has shown that their symptom burden were comparable with those of patients with advanced cancer [31–33]. Therefore, the World Health Organization has urged healthcare providers and policy makers to develop heightened awareness towards the palliative care needs of patients with advanced non-malignant conditions [34].

We acknowledge several study limitations that might affect the interpretation of findings. First, the sample size was small that undermines the power of the study to detect significant difference. Patient enrolment and willingness for receiving home visits were negatively affected because the study period clashed with social movement locally and the COVID-19 pandemic globally. The use of telecommunication evolved as the study progressed so as to maintain the service support to patients and family. Second, the participants were all cognitively sound and predominantly patients diagnosed with cancer. The study generalizability to non-cancer patients and patients with impaired mental capacity due to advanced conditions, such as delirium, could not yet be confirmed. Third, we did not have a control group for comparison in this cohort study because the project itself is a service improvement initiative. We can hardly ascertain the causal relationship between the intervention and the outcomes. Fourth, the data were only based on a PROM collected by the project team as part of the project services. The assessors were not blinded and the patients might have provided socially desirable responses to the project team members during the assessment. Moreover, assessing symptoms in the context of advanced disease on a monthly basis was relatively long. We did not arrange specific research personnel to conduct assessment separately due to resource limitation or increase the frequency of assessment to minimize disturbance and the response burden because the patients were generally frail. Fifth, we were unable to access the patients’ hospital records. Data about healthcare utilization were obtained from the self-report of patients or their family members.

### Implications for practice and research

The findings of this study has several implications for future practice and research. First, health and social care for community palliative care should be enhanced by involving multiple health disciplines to address the escalating care needs as the patients’ health condition deteriorates. For example, general practitioners and family physicians may adjust the medication for better symptom control, other allied health professionals may provide advices on dietary or physical exercise to maintain nutritional status or muscle strength. The wider scope of services might optimize the patients’ quality of life in the last phase of life [12]. Second, professional education should be strengthened for generalists to enhance their awareness and sensitivity toward the palliative care needs of patients, irrespective of diagnoses [1]. Third, additional research is warranted to examine the effects of community palliative care support on a larger and diverse group and the effects in the later phase of illnesses.

## Conclusions

This study evaluates the effects of a community-based model of palliative care for optimizing the quality of life of home-dwelling patients with advanced diseases. Their emotional symptoms and practical concerns are significantly improved in the first 2 months through a proactive, person-centered, and interdisciplinary approach delivered in a home care setting. This care model demonstrates the complementary role of non-government organization in filling the service gap for the integration of palliative care into primary care. Medical-social collaboration, telehealth, and palliative care education for generalists should be further strengthened to facilitate service accessibility.

## Data Availability

The datasets used and/or analysed during the current study are available from the corresponding author on reasonable request.

## Abbreviations

COVID-19: Coronavirus Disease 2019
IPOS: Integrated Palliative Care Outcome Scale
PROM: Patient reported outcome measure
